# Prevalence of obesity and hypertension among University students’ and their knowledge and attitude towards risk factors of Cardiovascular Disease (CVD) in Jeddah, Saudi Arabia

**DOI:** 10.12669/pjms.314.7953

**Published:** 2015

**Authors:** Mukhtiar Baig, Zohair J Gazzaz, Mamdooh A Gari, Haidar G Al-Attallah, Khaled S Al-Jedaani, Amjad TA Mesawa, Abdulrahman A Al-Hazmi

**Affiliations:** 1 Mukhtiar Baig, MBBS, PhD, Professor of Clinical Biochemistry, Faculty of Medicine, Rabigh, King Abdulaziz University, Jeddah, Saudi Arabia; 2 Zohair J Gazzaz, MBChB, PhD, Assisstant Professor of Medicine, Faculty of Medicine, Rabigh, King Abdulaziz University, Jeddah, Saudi Arabia; 3 Mamdooh A Gari, MSc., PhD Professor of Molecular Genetics, Faculty of Medicine, Rabigh, King Abdulaziz University, Jeddah, Saudi Arabia; 4 Haidar G Al-Attallah, Fifth Year Medical Students, Faculty of Medicine, Rabigh, King Abdulaziz University, Jeddah, Saudi Arabia; 5 Khaled S Al-Jedaani, Fifth Year Medical Students, Faculty of Medicine, Rabigh, King Abdulaziz University, Jeddah, Saudi Arabia; 6 Amjad TA Mesawa, Fifth Year Medical Students, Faculty of Medicine, Rabigh, King Abdulaziz University, Jeddah, Saudi Arabia; 7 Abdulrahman A Al-Hazmi, Fifth Year Medical Students, Faculty of Medicine, Rabigh, King Abdulaziz University, Jeddah, Saudi Arabia

**Keywords:** CVD, obesity, KSA, University students

## Abstract

**Objectives:**

To investigate the prevalence of obesity and hypertension among University students’ and their knowledge and attitude towards risk factors of cardiovascular disease (CVD) in Jeddah,: Saudi Arabia.

**Methods:**

A total of 610 male students were selected for present cross sectional study and their blood pressure (BP) and body mass index (BMI) was determined, other data was gathered through a questionnaire, and SPSS-16 was used for analyzing data.

**Results:**

Out of 610 students, 7.5% were hypertensive (systolic 2.6% and diastolic 6.3%) while the BMI of 51.6% was in the normal range, 29.8% were overweight and 10.7% were moderately obese and 7.9% were severely obese. Majority of the participants considered that smoking, increased fatty food intake, obesity, high BP, and increased LDL-cholesterol level, are the main causes of the CVD. Most of the participants agreed that one should know his BP, blood sugar, serum cholesterol and one should maintain normal body weight and should do regular exercise. They were also aware that healthy lifestyle could prevent CVD. However, majority of the participants were not practicing healthy lifestyle.

**Conclusion:**

A huge gap exists in the knowledge, attitude and practice regarding risk factors of CVD among the university students.

## INTRODUCTION

The WHO global status report on noncommunicable disaeses (2010) mentioned that CVDs are the top most reason of death worldwide and in 2008, around 17.3 million people passed away because of CVDs, representing 30% of all deaths worldwide.^1^ A report by Lim et al., (2012) pointed out that 16.5% of all deaths each year (9.4 million deaths), are because of the hypertension and this includes strokes and coronary heart disease major contributing cause of death 51% and 45% respectively.^2^ It is estimated that by the 2030, the number of death because of CVDs will reach to 23.3 million. 3 Most of the CVDs can be averted by tackling associated risk factors like physical inactivity, use of tobacco, diabetes mellitus, unhealthy diet, obesity, raised lipids, and high BP.^3^

Al-Rethaiaa et al., reported that among college students, the prevalence of overweight and obesity was 21.8% and 15.7%, respectively.^4^ Another study found 40% occurrence of age-adjusted obesity in KSA while incidence of CAD and hypertension was 6.9% & 32.6%, respectively.^5^ A recent study described that 28.3% male medical students in Jeddah were overweight, 18.9% were obese, and 9.3% were hypertensive.^6^

Numerous studies are available about the perception regarding knowledge, attitudes and practices on CVD risk factors but most of the studies related with the women perception^7^-^8^, only few studies available about the perception of young people. It is imperative to understand the young generation’s existing knowledge, attitudes and practices about CVD risk factors for initiating the preventive efforts. Therefore, this study was designed to identify the prevalence of obesity and hypertension among University students’ and their knowledge and attitude towards risk factors of CVD in Jeddah, Saudi Arabia.

## METHODS

The present cross sectional study was accomplished at the Rabigh and Jeddah campuses of King Abdulaziz University, Jeddah, Saudi Arabia, in the year 2014. The calculated sample size for this study was 246 by using the following formula^9^,

n=Z_1-α/2_^2^ p(1-p)/d^2^

n= the minimum sample size, Z_1-α/2=_ Is standard normal variate (at 5% type 1 error (*P*<0.05) it is 1.96, p= Proportion of of obesity described by previous study (20%)^10^, d=absolute error or precision (5%). Almost double sample size was included to consider non-participant rate.

A total of 610 young male students participated in the study from Rabigh and Jeddah campuses. Data was collected on a self-administered questionnaire in Arabic language, which was prepared with the help of previously published related studies. Data was gathered on age, marital status, and physical activities through a questionnaire; additionally, it also had knowledge, attitudes and practices questions about risk factors of CVD. Blood pressure, weight and height of the participants were measured. The cut-off points to determine systolic or diastolic hypertension were based on the seventh JNC classification of hypertension (≥140/90 mmHg).^11^ Ethical approval was taken from the institute’s ethical review committee and informed consent was taken from all the participants.

***Statistical Analysis:*** We ranked knowledge variable from zero to 2 points, and zero was for negative answer. Attitude and practice variables were judged on the basis of three-point Likert type scale and for each question, the lowest point was zero and the highest 2. The total score was labeled good on the basis of those positive responses of the participants about the particular question. The data was analyzed on SPSS 16.

## RESULTS

The mean age of the respondents was 22.40±3.90, out of 610 subjects 166 (27.2%) were married, and 444(72.8%) were unmarried. The majority of the respondents 414 (67.9%) agreed that heart attack is a major problem and 461 (75.6%) considered it as a preventable problem and 66 (10.8%) considered bad luck as a cause of heart attack (not shown in table).

There were 46 (7.5%) students hypertensive (systolic 2.6% and diastolic 6.3%) while the BMI of 315 (51.6%) was in normal range (BMI < 24.9), 182 (29.8%) were overweight (BMI 25-29.9) and 65(10.7%) were moderately obese (BMI 30-34.9) and 48(7.9%) were severely obese (BMI >35) (Table-I). The respondents’ knowledge, attitude and practice results are shown in Table II, III and IV.

**Table-I:** Blood pressure and BMI of the participants.

Parameter	N (%)
*Blood pressure*	
Normotensive	564(92.5)
Hypertensive	46(7.5)
-Systolic	2.6%
-Diastolic	6.3%
*BMI (Kg/m^2^)*	
- <24.9	315(51.6)
- 25-29.9	182(29.8)
- 30-34.9	65(10.7)
- >35	48(7.9)

N=number of participants.

**Table-II:** Knowledge items with mean score (SD) and percentage (%) for good knowledge towards CVD (n=610).

Item	Mean (SD)	Good knowledge N (%)
*Knowledge on CVD risks*		
Smoking	1.78 (0.55)	513(84.1)
High blood pressure	1.37 (0.87)	388(63.6)
Obesity	1.65 (0.70)	475(77.9)
High LDL cholesterol	1.34 (0.90)	390(63.9)
Increasing age (>55years)	0.97 (0.73)	153(25.1)
Stress	1.16 (0.89)	297(48.7)
Family history of heart disease	1.01 (0.86)	226(37)
Chronic renal failure	0.66 (0.77)	109(17.9)
Diabetes mellitus	0.90 (0.83)	185(30.3)
Increased fatty food intake	1.65 (0.65)	455(74.6)
Increased use of salt	1.01 (0.88)	237(38.9)
Excessive use of organ meat	0.80 (0.84)	166(27.2)
Sedentary life style	0.87 (0.63)	88(14.4)
Waist circumference >40 inches	0.57 (0.77)	105(17.2)
BMI >30	0.71 (0.85)	156(25.6)

SD=Standard deviation.

**Table-III:** Attitude & practice items with mean score (SD) and percentage (%) for positive attitude & practice towards CVD (n=610).

Item	Mean (SD)	Good attitude N (%)
*Attitude Items*		
Should know blood pressure level	1.79(0.49)	502(82.3)
Should know cholesterol level	1.76(0.50)	486(79.7)
Should know blood sugar level	1.82(0.46)	517(84.8)
Should maintain normal body weight	1.88(0.38)	551(90.3)
Willing to exercise regularly	1.84(0.44)	531(87.0)
Willing to maintain healthy lifestyle	1.78(0.51)	503(82.5)
Try to reduce sugar intake	1.59(0.61)	400(65.6)
Try to reduce fat intake	1.71(0.54)	460(75.4)
*Practice Items*		
Exercise more than 20 minutes 3days/week	0.88(0.71)	120(19.7)
Play outdoor games daily/thrice in a week	0.97(0.73)	155(25.4)
Eat outside home	0.62(0.55)	21(3.4)
Use more than 3 teaspoon salt/day	1.11(0.72)	198(32.5)
Avoid fatty foods	0.86(0.62)	79(13.0)
Maintain normal weight	0.67(0.67)	69(11.3)
Try to reduce stress	0.44(0.61)	39(6.4)
Avoid smoking	0.5(0.72)	82(13.4)
Visit doctor for advice	1.39(0.68)	304(49.8)
Eat fish thrice/week	1.18(0.64)	189(31)
Gain knowledge about CVD through mass media or electronic	0.93(0.65)	107(17.5)
Try to prevent CVD	1.08(0.70)	124(20.3)

SD=Standard deviation.

Proportion of positive attitude and practice who answer “agree” for attitude that they should do & answer “always” for practice that they should adopt.

**Table-IV:** Mean of Knowledge, attitude and practice.

	Mean(SD)	% Good knowledge, attitude, practice
Knowledge	1.10 (0.35)	43.09%
Attitude	1.77 (0.31)	80.95%
Practice	0.89 (0.27)	20.30%

SD=Standard deviation.

## DISCUSSION

In present era, overweight and obesity are considered as an escalating pandemic. The measurement of BMI is considered as a representative of obesity and it is one of the recognized predisposing reason of CVD. In current study about 49% university students did not have normal BMI (30% were overweight, 11% were moderately obese and 8% severely obese). Our findings synchronize with results of Ibrahim et al., that described a similar rate of overweight and obesity among male medical students^6^ and similar to another USA study that reported 33% of prevalence of overweight and obesity among university students in USA.^12^

Al-Rukban reported that the overweight and obesity prevalence amongst male adolescents in Riyadh, Saudi Arabia was 13.8% and 20.5% respectively.^10^ Furthermore, a study done in Eastern Saudi Arabia found that prevalence of overweight and obesity among Saudi male adolescents was 14.1% & 16.7% respectively.^13^ The results of the present and previous studies have reported that the prevalence of obesity among young generation in KSA is at higher level, hence, it needs intervention on priority bases. Sabra et al., suggested that the lack of physical activity and rapidly increasing tendency of consuming fast food is responsible for high incidence of overweight and obesity among young generation.^13^

In present study, 7.5% of the students having hypertension (≥140/90 mmHg); 2.6% had systolic and 6.3% had diastolic hypertension and hypertension is a well-known important risk factor for heart disease and stroke. These results are consistent with Ibrahim et al.,^6^ who reported 9.3% prevalence of hypertension among medical students in Jeddah while 3.7% had systolic and 7.9% had diastolic BP.

A study at King Fahd University in Dammam city stated higher rates of systolic and diastolic hypertension (13.8%% and 3.7%, respectively) among male students.^13^ Al-Almaie, reported similar prevalence of hypertension.^14^ Al-Daghri et al., found incidence of hypertension was 32.6% in Riyadh region of KSA.^5^ The prevalence of hypertension among young adults needs urgent attention and further evaluation because of the grave consequences of hypertension and because secondary hypertension is likely in this age group. Lee & Cooper suggested that for CVD, the hypertension is an important amendable risk factor.^15^

### Knowledge and attitude on cardiovascular disease

In present study, respondents knowledge was not good but they had good attitude. They were unaware about few important factors implicated in the CVD, like stress, diabetes mellitus, chronic renal failure, increased use of salt, excessive use of organ meat such as liver, kidney and brain, sedentary life style, waist circumference >40 inches, BMI >30.

Smoking was identified for CVD risks by majority of the students (84.1%) followed by obesity, (77.9%) and increase intake of fatty food (74.6%). However, only 14.4% knew that sedentary life style is one of the risk factors. These results are in agreement with several studies.^7^,^8^ So there is need to enhance the knowledge of the young generation regarding CVD.

Most of the participants agreed that they should maintain their normal body weight and do regular exercise. Furthermore, they agreed that they should know their cholesterol, BP and blood sugar level. They were also willing to maintain healthy life style. They wanted to reduce fat and sugar intake. These results are consistent with a study^9^ that described the reason of participants significantly positive attitude towards risk factors could be their awareness about healthy lifestyle as promoted in the mass media. Another reason could be the increasing advertisement about weight reducing equipments, medicines, exercise machines and physical fitness programs which have created awareness among masses.

The present study found 43% of mean score of knowledge. A study in Pakistan also reported similar mean score of knowledge (42%).^16^ Vanhecke et al., in their study described that adolescents in the USA don’t have sufficient knowledge about the risk of CVD and furthermore, they do not perceive themselves at risk for CVD.^17^

In current study, sedentary life style, increased waist circumference, chronic renal failure showed the low score (14.4%, 17.2%, 17.9% respectively). Therefore, these factors need more attention by doctors and health policy makers. In this region sedentary lifestyle and obesity is more prevalent and unfortunately, younger generation do not consider that these are associated with CVD. Recently, Tedesco et al., reported that 89.4% and 74.7% respondents correctly identified smoking and high cholesterol level as risk factors for CVDs, while only 26.5% of the participants were able to correctly recognize the main CVDs risk factors.^18^

We strongly suggest that there is need to improve younger generation knowledge regarding risk factors of CVD and to convince them to bring changes in their life style and dietary pattern to avoid this fatal problem.

Although, 87% of the subjects were willing to exercise regularly, implying their high positive attitude, but only 19.7% exercised less than 3 times a week. Mazloomy et al., found 91% positive attitude, and 74% practice in their study.^19^ Hence, we strongly feel that there is need to educate our younger generation about the importance of healthy life style. Sabra et al., observed among Damam University students that many students (>25%) do not carry out any type of physical exercise.^13^

Another study showed that 71% of youth and 60% of Saudi children have lack of physical activity.^20^ Indeed, television viewing, computer, & videos games are playing very important contributing role in propagation of this physical inactivity epidemic.^20^ Lack of physical activity is considered a main contributing factor for CVD.^1^

In present study, only 17.5% students gained knowledge about CVD from electronic media. This could be because of the reason that young generation is not watching such informative, educational and health issues related programs. Another reason could be that the electronic media is not transmitting appropriate information about the CVD and not giving sufficient time to this important issue.

### Practice on cardiovascular disease

The present study observed suboptimal practicing (20%) among the participants. A study also observed that overall practice regarding CVD risk reduction behaviour was suboptimal in men and women in USA.^21^

One of the reason for poor practice could be the lack of awareness about factors causing CVDs like majority of the participants didn’t know that stress, diabetes mellitus, sedentary life style, increased use of salt, excessive use of organ meat such as liver, kidney and brain are risk factors for CVD. In our study, only 19.3% do regular exercise more than 20 minutes 3days/week which is higher than a study by Rosediani et al., (13.4 %)^9^ and lower than another study (63%). 21 There is possibility that the lack of practicing CVD risk reduction behavior would aggravate the condition and incidence of CVDs would enhance enormously in the community.

In our respondents, the attitude about risk factors of CVDs was good but practice was poor. One of the reasons could be that it is always very difficult to change the lifestyle pattern. In KSA, people like eating sugary items in many forms like chocolate, confectionary, juices, cakes. Moreover, young generation is very fond of eating fast food and drinking soft drinks. So people’s dietary pattern lead them to become overweight and obese and they are also more prone to develop CVD.

### Limitations

There are few limitations to our study like, it was a cross sectional study based on convenient sampling technique and the knowledge, attitude and practice about CVD were self-reported and only BMI was taken as an indicator of obesity.

## CONCLUSION

Almost half of the participants of this study were either overweight or obese and 7% were hypertensive. A huge gap exists in the knowledge, attitude and practice among the sample of young Saudi population regarding risk factors of CVD.

## RECOMMENDATIONS

A large scale awareness program should be initiated from health ministry on the electronic and print media.
